# Adhesive and Magnetic Properties of Polyvinyl Butyral Composites with Embedded Metallic Nanoparticles

**DOI:** 10.3390/s21248311

**Published:** 2021-12-12

**Authors:** Tatyana V. Terziyan, Alexander P. Safronov, Igor V. Beketov, Anatoly I. Medvedev, Sergio Fernandez Armas, Galina V. Kurlyandskaya

**Affiliations:** 1 Institute of Natural Sciences and Mathematics, Ural Federal University, 620002 Ekaterinburg, Russia; tatyana.terzyan@urfu.ru (T.V.T.); alexander.safronov@urfu.ru (A.P.S.); beketov@iep.uran.ru (I.V.B.); 2 Pulsed Processes Laboratory, Institute of Electrophysics UB RAS, 620016 Ekaterinburg, Russia; medtom@iep.uran.ru; 3 SGIKER, Basque Country University UPV/EHU, 48940 Leioa, Spain; sergio.fernandez@ehu.eus; 4 Department of Electricity and Electronics, Basque Country University UPV/EHU, 48940 Leioa, Spain

**Keywords:** polyvinyl butyral, electric explosion of wire, metallic nanoparticles, magnetic nanoparticles, polymer filled composites

## Abstract

Magnetic metallic nanoparticles (MNPs) of Ni, Ni82Fe18, Ni50Fe50, Ni64Fe36, and Fe were prepared by the technique of the electrical explosion of metal wire. The average size of the MNPs of all types was in the interval of 50 to 100 nm. Magnetic polymeric composites based on polyvinyl butyral with embedded metal MNPs were synthesized and their structural, adhesive, and magnetic properties were comparatively analyzed. The interaction of polyvinyl butyral (supplied as commercial GE cryogenic varnish) with metal MNPs was studied by microcalorimetry. The enthalpy of adhesion was also evaluated. The positive values of the enthalpy of interaction with GE increase in the series Ni82Fe18, Ni64Fe36, Ni50Fe50, and Fe. Interaction of Ni MNPs with GE polymer showed the negative change in the enthalpy. No interfacial adhesion of GE polymer to the surface of Fe and permalloy MNPs in composites was observed. The enthalpy of interaction with GE polymer was close to zero for Ni95Fe5 composite. Structural characterization of the GE/Ni composites with the MNPs with the lowest saturation magnetization confirmed that they tended to be aggregated even for the materials with lowest MNPs concentrations due to magnetic interaction between permalloy MNPs. In the case of GE composites with Ni MNPs, a favorable adhesion of GE polymer to the surface of MNPs was observed.

## 1. Introduction

Polyvinyl butyral (PVB) is a random terpolymer mainly composed of vinyl alcohol and vinyl butyral with relatively small amounts of vinyl acetate. A terpolymer is a copolymer in which two or more chemically distinct monomer units are alternating along linear chains in the irregular way. PVB is a colorless, amorphous thermoplastic resin [1], which is widely used in technological applications such as automotive laminated glass, paints, and adhesives due to its excellent flexibility, ability to form coatings in the film shape, good adhesion properties, and excellent UV resistance. Easy wettability and compatibility with various polar compounds (such as phenols, epoxies, isocyanates, etc.) make PVB an excellent candidate to be used in many functional applications. On its basis, composite materials with inorganic fillers of various chemical nature can be fabricated. Thus, the development of shape memory materials containing graphene oxide [2], photoactive materials with improved mechanical and heat-conducting properties with particles of TiO_2_, CdS and other ceramic fillers can be mentioned [3,4,5].

Fillers of the metallic nature are also considered to be components of PVB-based systems. For instance, Angappan et al. [6] described the preparation of a composite based on PVB and a core-shell filler, where a thin nickel layer was used as a coating. The material was designed as a lightweight broadband microwave absorber for different applications. Metallic particles are often called “zero-valent” particles to distinguish them from metal oxide particles. In pure metals, atoms are zero-valent, while in oxides metal atoms have a positive oxidation number according to their valency. Although the term “metallic” presumably means elemental (zero-valent) metal, still in some biomedical references (and applications) it is sometimes attributed to metal oxides also.

In addition, PVB is a component of binders, in particular, a component of “GE varnish” (being not an abbreviation but a commercial product label) widely used to thermally anchor wires at cryogenic temperatures. It has fast track time and can be both air-dried or baked. Other important features of this adhesive are electrical insulating properties at cryogenic temperatures, suitable properties of a calorimeter cement, compatibility with a wide variety of materials, including cotton, nylon glass tapes, mica products, polyester products, vinyl products, plastics, and many others. In the research laboratories, GE varnish was widely used in the studies on the magnetic and microwave characteristics of the wide variety of materials [7,8,9,10]. In fact, when measuring the properties of magnetic particles, a filled polymer composition is usually prepared, where the particles under study act as a dispersed filler in a GE-varnish polymer matrix.

One of the major structural parameters of a polymer/filler composite material is the uniformity of the distribution of particles in the polymer matrix. However, it is difficult to achieve uniform distribution. Here it is important to mention that the techniques of characterization of the filler distribution are not developed yet at a satisfactory level. There are different parameters of the fillers themselves contributing to the uniformity of the filler distribution inside the composite. First, it is filler chemical composition and the shape of the filler particles (spherical, cubic, rod-like etc.). For the case of magnetic fillers, the interaction between the filler particles plays crucial role preventing their de-aggregation during polymer/filler composite fabrication. Second, practically all kinds of available fillers, especially those that can be obtained in the large quantities, have distribution of the shapes and sizes of the elements. This obstacle adds extra difficulties in the control of the uniformity of the distribution of particles in the polymer matrix. For example, it is well known that magnetic behavior of the ferrofluid critically depends on the presence of even a few particles of the large size in the ensemble [11].

Particle distribution affects thermal and electrical characteristics [5], mechanical, dielectric and microwave properties of composites based on PVB [3,7]. The uniformity of the particle distribution inside the composite is also affected by the adhesive interaction between the polymer matrix and the dispersed filler. The higher the adhesion of the polymer to the surface of the particles, the greater the likelihood of their disaggregation with distribution in the form of individual particles. In this regard, the use of GE varnish as a binder for fixing a certain distribution of magnetic filler requires an understanding of the degree of adhesive interaction between the components of the GE varnish and the magnetic particles.

When we refer to the bulk ferromagnets in thin film state, one of the most studied systems is the system of iron-nickel alloys starting from pure nickel and up to the pure iron [12,13,14]. The saturation magnetization evolution, magnetic anisotropy features, magnetic permeability and magnetostriction changes were widely discussed and comparatively analyzed [15,16,17]. Apart from the theoretical interest, this system is widely used in many practically important devices [18,19]. However, nanostructured FeNi alloys in the shape of nanoparticles and filled composites on their basis were studied to lesser extent and there is a gap or absence of the understanding to what extent the results obtained in the case of FeNi system in the bulk thin film state can be applicable to the MNPs related cases.

In this work, we have studied the structure, magnetic properties and interactions at the interface of the composite films based on polyvinyl butyral terpolymer (GE varnish) and magnetic nanoparticles of nickel, iron, and FeNi of various compositions.

## 2. Materials and Methods

GE varnish was a commercial product GE-7031-CT Thermal Varnish (CRYO-Technics, Büttelborn, Germany). It was a viscous liquid brownish 25% solution of a polymer in mixed organic solvent. GE varnish was used as received for the preparation of polymeric composites with embedded zero-valent metallic nanoparticles.

To minimize the contribution of the shape variation of the particles of the filler highly productive electrophysical technique of the electrical explosion of metal wire (EEW) was used for the synthesis of magnetic metallic nanoparticles (MNPs) [20]. The method itself was known long ago [21] but in recent years special attention was focused on it as a technique for MNPs fabrication [22]. Special advantage of the technique is the fabrication of the particles of close to spherical shape. MNPs were synthesized at the Laboratory of Pulsed Processes of the Institute of Electrophysics UB RAS via the electrical explosion of metal wire in argon. In EEW method a metallic wire is evaporated by a high voltage discharge, and the vapors condense in gas phase giving spherical non-agglomerated nanoparticles. The details of the method and the description of the experimental EEW setup can be found elsewhere [20,22]. Using EEW method magnetic nanoparticles of Ni, Ni82Fe18, Ni64Fe36, Ni50Fe50, and Fe were synthesized. The batches of Fe and Ni MNPs were approximately 500 g each and the batches of NiFe alloys were approximately 100 g each.

To prepare GE/MNPs composites, first, the weighted portion of MNPs of each type was grinded in the agate mortar with the addition of isopropanol to make a homogeneous suspension of MNPs. Then the weighted amount of GE varnish was added and vigorously mixed with the MNPs suspension. The resulted slurry was then cast onto the polished glass and left at ambient conditions for the evaporation of the solvent. The dried film of GE/MNPs composite was mechanically separated from the glass substrate and kept in a thermostat at 80 °C up to the reaching of the state of the constant weight when all residual solvents were eliminated. The wide range of polymer/MNPs compositions was selected to characterize extensively the enthalpy of adhesion of polymeric matrix to the surface of MNPs both at low MNPs content and at the highest obtainable content. In this respect mechanical properties of the compositions were not considered but they are certainly important for any practical application, e.g., for microwave adsorption. In this respect, compositions with MNPs content below 70% are preferable as they become brittle at the higher content of MNPs. Even so, for the present study in selected cases the MNPs content up to 88% was considered.

Transmission electron microscopy (TEM) images were obtained using a JEOL JEM2100 microscope operated at 200 kV (JEOL Ltd., Tokyo, Japan). The particles were dispersed in isopropanol under ultrasonic treatment and the resulted suspension was placed onto carbon coated copper grids and evaporated at ambient conditions. X-ray diffraction (XRD) studies were performed using a BrukerD8 Discover (Bruker, Billerica, MA, USA) instrument with Cu-Kα radiation (wavelength λ = 1.5418 Å), a graphite monochromator for a diffracted beam and a scintillation detector. Diffractograms were refined by Rietveld algorithm using TOPAS 3.0 program installed in the XRD instrument. The specific surface area of MNPs was measured by low-temperature adsorption of nitrogen using Micromeritics TriStar3000 automatic sorption analyzer (Micromeritics, Norcross, GA, USA).

Magnetic measurements of the magnetization value as a function of the applied magnetic field *M*(*H*) (hysteresis loops) were performed at the room temperature by a vibrating sample magnetometer (Cryogenics Ltd. VSM, London, UK). Even though complete saturation was not always achieved in the field of 1.8 kOe, we designate the meaning of saturation magnetization (M_s_) to the value of magnetization in *H* = 1.8 kOe. Both *M_s_* and coercivity (*H_c_*) value were calculated from the *M*(*H*) hysteresis loops. The MNPs were measured in non-magnetic capsule (up to 5 mg of the sample weight) and GE/MNPs composites (up to 15 mg of the weight of the sample composite) were measured for in-plane configuration of the film.

Fourier transform infra-red (FTIR) spectra of composite films were obtained in the 4000–500 cm^−1^ range of wavenumbers using a Nicolet 6700 FTIR spectrometer equipped with an ATR add-on (Thermo Fisher Scientific, IN, USA)

Calorimetric measurements were performed at 25 °C using Calvet 3D differential microcalorimeter DAK-1-1 (EPSI, Chernogolovka, Russia) with ampoule cells. A portion (20–90 mg) of GE composite or air-dry MNPs was put into a thin glass ampoule ca.5 mL in volume and dried in an oven to a constant weight. After that the ampoule was sealed and placed in sliding holder, mounted to the top of a stainless steel tubular cell (7 cm^3^). The cell was filled with 4 mL of isopropanol. Two assembled cells were positioned in the ports of microcalorimeter and it was thermostated at 25 °C for 2–3 h until the drift of the baseline fell below 0.01 mW in 30 min. Then the experiment in one of the cells (working cell) was initiated by breaking the glass ampoule in the solvent (isopropanol). The other cell was a reference. Heat evolution curve in the working cell was recorded for ca 60–90 min until the initial baseline was reestablished. The time dependence of heat flux was integrated using software program giving the enthalpy of dissolution for the sample in the ampoule. Then the experiment in the second cell was performed in a similar manner. Typical values of heat effects were within the range 0.1–5.0 J, depended on the load in the ampoule. The relative error of measurements was 5% for the heat effects ranging from 0.1 to 0.5 J and 2.0% for the heat effects in the 0.5–5.0 J range.

In addition, the structure of selected filled composites was studied by scanning electron JEOL JSM-640 microscope (JEOL Ltd., Tokyo, Japan) working at 20 kV accelerating voltage and equipped with energy dispersive X-ray (EDX) fluorescent detector for elemental analysis. As before [23] to avoid the charging of the non-conducting polymer surface, about 20 nm carbon layer was deposited onto the composite surface.

## 3. Results

**Figure 1:**
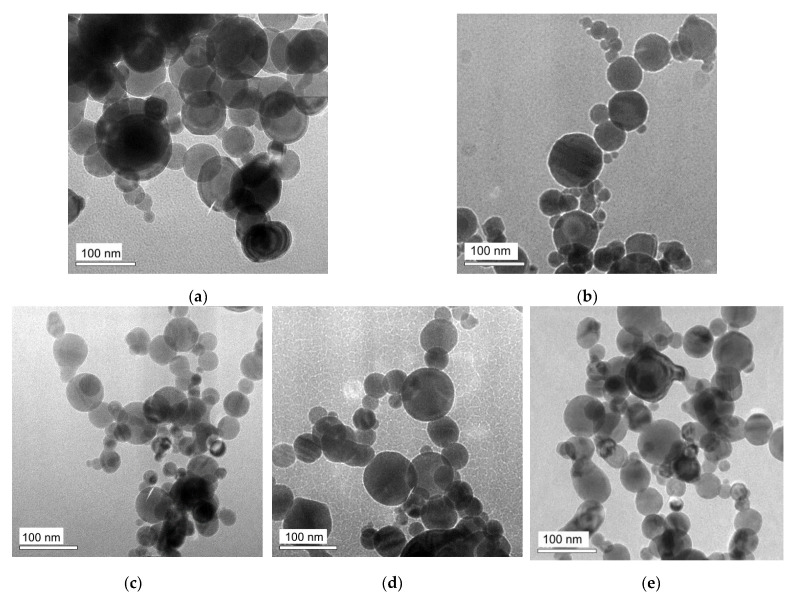
TEM images of zero-valent metal EEW MNPs of different types. (**a**)—Fe, (**b**)—Ni50Fe50, (**c**)—Ni64Fe36, (**d**)—Ni82Fe18, (**e**)—Ni.

Figure 1 shows transmission electron microscopy images of synthesized zero-valent metallic spherical EEW MNPs with different content of iron and nickel. As the shape of the MNPs was close to the spherical one and therefore their diameter was selected as characteristic geometrical parameter.

The average apparent characteristic diameters (*d_S_*) in for the batches of all types were calculated based on the value of specific surface area according to following equation [24]:

(1) $$d_{S}=\frac{6000}{\rho S_{sp}}$$

Here *S_sp_* is the specific surface area of MNPs, which is conventionally measured using low-temperature adsorption of nitrogen (Brunauer–Emett–Teller (BET) method [16]); *ρ*—is the crystallographic density of MNPs. The calculated values of the apparent characteristic diameters of the MNPs are given in Table 1. One can see that all obtained batches can be considered to be the MNPs. The highest *d_S_* value corresponded to the iron MNPs and the smallest one to the nickel MNPs. section may be divided by subheadings. It should provide a concise and precise description of the experimental results, their interpretation, as well as the experimental conclusions that can be drawn.

According to XRD data analysis phase composition of MNPs corresponded to solid solutions based on cubic crystal structure. Their specific parameters are given in Table 1 as well as the values of the saturation magnetization and the coercivities.

General comparative analysis of the *d_S_* and *M_s_* values shows good correlation between their values, and it is in accordance with the existing understanding of magnetic behavior of nanoparticles of these sizes and compositions [23,25,26,27]. In all cases the saturation magnetization was lower in comparison with bulk *M_s_* values [28,29] with the difference of 15 to 25%. However, as to expect, the highest *M_s_* was observed for iron MNPs and the lowest saturation magnetization was obtained for Ni MNPs.

The reduction of the value of the saturation magnetization in comparison with the bulk state can be assigned to different effects. The first one is the strongly pyrophoric features of EEW MNPs and the need for their surface passivation prior to the exposure to the atmosphere. In the previous studies [11,23,30] we have shown that the passivation oxide layer has a thickness of a few nm.

However, for the MNPs with *d_S_* in the range of about 50 to 100 nm such a layer with lower magnetization can cause a reduction up to 15% of the total magnetization value (depending on the size of the MNPs). The second reason of the reduction of the saturation magnetization value is related to the concept of nanoscaling laws [31]. In the spherical MNPs at least three surface layers are not contributing to the ferromagnetic response not having the sufficient number of the nearest neighbors. For instance, in pure a-Fe one can obtain the reduction up to 10% in comparison with value of the bulk iron. Both abovementioned reasons for the M_s_ were given without taking into account the existence of the MNPs size distribution which makes the analysis even more difficult. Even so, fabricated batches of the metallic MNPs were used for comparative analysis of their adhesive and magnetic properties of GE varnish (polyvinyl butyral)-based composites.

Unfortutately, narrowing of the distribution of metallic MNPs is not achievable using conventional separation techniques such as filtering of separation. The basic reason for this is strong aggregation of metallic MNPs in their suspensions. Suspensions of metallic MNPs do not contain individual particles. These features of them were discussed in our recent paper by Shankar et al. [32]. Theoretical consideration by the extended DLVO approach favored strong magnetic interactions as a major reason for aggregation. Therefore, the batches of metal MNPs are to be used as synthesized.

**Figure 2:**
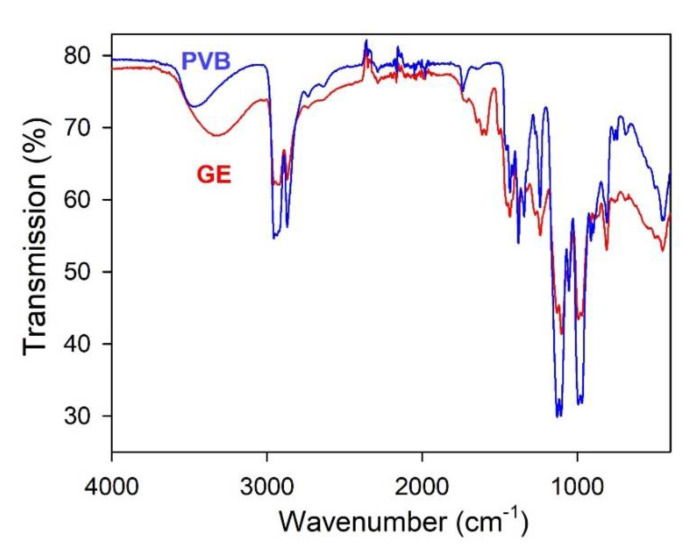
FTIR spectra of poly(vinyl butyral) (PVB) and GE polymer.

GE varnish is a multi-component industrial product. Figure 2 presents FTIR spectrum in the range of wave numbers from 400 up to 4000 cm^−1^, which was obtained aiming to clarify the chemical composition of the available product. The spectrum refers to the polymeric residue, which was obtained after the evaporation of the solvent from GE varnish. To mark it out further we will denote this polymeric residue as GE polymer or simply GE. The strongest bands in the GE spectrum were: a peak at 3370 cm^−1^, which was attributed to the stretching of OH group, peaks at 2955 cm^−1^ and 2869 cm^−1^ due to the stretching of C-H bonds in aliphatic CH_3_, CH_2_ and CH groups, and a peak at 1712 cm^−1^ due to the stretching of carbonyl group. The peaks at 1434 cm^−1^ and 1129 cm^−1^ are attributed to the vibrations of CH_2_ and C-O-C groups. Identification of the spectrum gave the 93% fit for poly(vinyl butyral) [33]. The difference in GE polymer and PVB IR spectra were observed only in the range of wave numbers 3600–2600 cm^−1^ corresponding to the vibrations of hydroxyl groups (Figure 2).

The frequency of OH group stretching in individual molecules is around 3600 cm^−1^ [25]. The broad bands in Figure 2 are shifted to lower frequencies for both GE and PVB. It means that the hydroxyl groups of polymers are linked by hydrogen bonds. In the case of the GE polymer, the shift is observed to a greater extent. FTIR spectrum of the GE shows very wide absorption peak at 3320 cm^−1^ due to self-associated OH groups.

The close identity of FTIR spectra for GE polymer and for PVB, except for the shape of the OH peak, indicated that both PVB and GE have the same basic chemical structure and differ in the degree of self-association by hydrogen bonds.

Interfacial interactions of GE polymer with zero-valent metallic MNPs were evaluated by microcalorimetry. For obvious reasons it is not possible to mix directly in a calorimeter solid nanoparticles with a solid polymer.

Therefore, the enthalpy of interaction among nanoparticles and polymeric matrix are calculated using thermochemical cycle [34,35] based on the measurable heat effects of several appropriate processes, which give in combination the desired enthalpy change. In the case of a GE polymeric composite the enthalpy of formation (Δ*H_comp_*) refers to the process:

GE + *MNPs* => GE/*MNPs composite* + Δ*Hcomp* (2)

As the components of a polymeric composite do not dissolve in each other, Δ*H_comp_* solely depends on the interfacial interaction between solid particles and GE polymeric matrix. Note that Δ*H_comp_* is a function of the content of MNPs in composite and it should better be written as Δ*H_comp_*(*ω*), with *ω* standing for the weight fraction of MNPs in GE composite.

The combination of processes that comprise Equation (2) and can be performed in calorimetric cell is given below:

GE + isopropanol => GE solution + Δ*HGE* (3)

MNPs + isopropanol => MNPs suspension + Δ*HMNPs* (4)

GE solution + MNPs suspension => MNPs suspension in GE solution + Δ*H_mix_* (5)

GE/MNPs composite + isopropanol => MNPs suspension in GE solution + Δ*H_dis_*(*ω*)** (6)

Δ*H_GE_* is the enthalpy of dissolution of GE; Δ*H_MNPs_* is the enthalpy of wetting of MNPs; Δ*H_mix_* is the enthalpy of mixing suspension with solution; Δ*H_dis_*(*ω*)** is the enthalpy of dissolution of a composite with weight fraction of MNPs equal to ω.

The combination of steps is: (2) = (3) + (4) + (5) − (6), and it gives the following equation for the enthalpy of composite formation:

Δ*H_comp_*(*ω*) *= ω* × Δ*H_GE_* + (1 − *ω*) × Δ*H_MNPs_ +* Δ*H_mix_* − Δ*H_dis_*(*ω*)** (7)

Typically, the term Δ*H_mix_* is much lower than others. It falls within the experimental error of calorimetric measurements and can be neglected.

**Figure 3:**
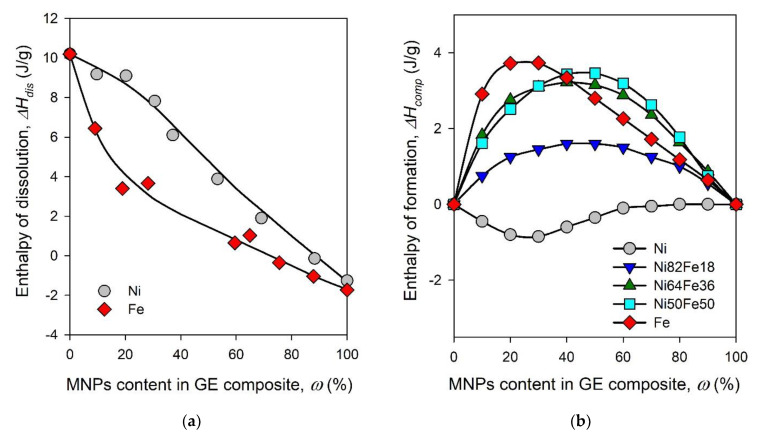
Concentration dependence of the enthalpy of dissolution in isopropanol for GE composites with Ni and Fe MNPs (**a**). Concentration dependences of the enthalpy of formation for GE/MNPs composites (**b**). T = 25 °C.

Figure 3a shows the typical view of concentration dependences of the enthalpy of dissolution for polymeric composites based on GE polymer with embedded MNPs. All experimentally measured thermal effects are expressed in Joules per gram of the samples used in the calorimetric experiment. Point on the left axis corresponds to the value of Δ*H_GE_*, which was positive for the dissolution of GE in isopropanol. Points at the right axis correspond to Δ*H_MNPs_* values for Ni and Fe MNPs. These values are small and negative. All other points in the plot correspond to Δ*H_dis_*(*ω*) of composites. These data were used for the calculation of the enthalpy of formation for GE/MNPs composites in the entire range of nanoparticles content. Concentration dependences are given in Figure 3b. According to Figure 3b, the enthalpy of formation of the GE composites with Fe and all marks of NiFe MNPs is endothermic over the whole range of compositions, i.e., during the formation of the composites the heat was absorbed. Concentration dependence of the enthalpy of formation for GE/Ni composite is negative over the entire composition range. Concentration dependences for GE composites with NiFe MNPs lay between the plots for GE/Ni and GE/Fe composites.

The reason for the existence of the concentration dependence of the enthalpy of formation of polymeric composites is not trivial, since the components in the composite do not dissolve in each other. In this case, the enthalpy change is due to the interfacial adhesion between polymer matrix and the surface of solid MNPs. Polymeric molecules are large, they can form a variety of conformations at the interface [27]. Therefore, the thickness of the interfacial layer at the solid surface in contact with polymer is likely extended compared to the interface with simple liquids. Thus, the enthalpy of adhesion of a polymer to a solid surface depends on the degree of saturation for interfacial polymeric layer, which is a function of the content of solids in polymeric composite. In Figure 3b Δ*H_comp_* is zero at *ω* = 0, i.e., for individual GE, because there are no interfacial layers in it. As the content of MNPs increases, the total area of the interface between MNPs and GE increases as well and so do the absolute values of Δ*H_comp_*. At a certain content of MNPs in a composite all polymeric molecules would be involved in the formation of interfacial layers, and absolute values of Δ*H_comp_* would reach their maximum. At a level of MNPs content above this threshold the interfacial layers would become progressively unsaturated and it would diminish the enthalpy of composite formation down to zero at *ω* = 0 that corresponds to individual MNPs. Therefore, we might consider the maximum absolute value of the enthalpy of composite formation in Figure 3b as an indicative measure for the intensity of interfacial interactions (Δ*H_int_*) of GE polymer with the surface of a certain type of MNPs.

**Figure 4:**
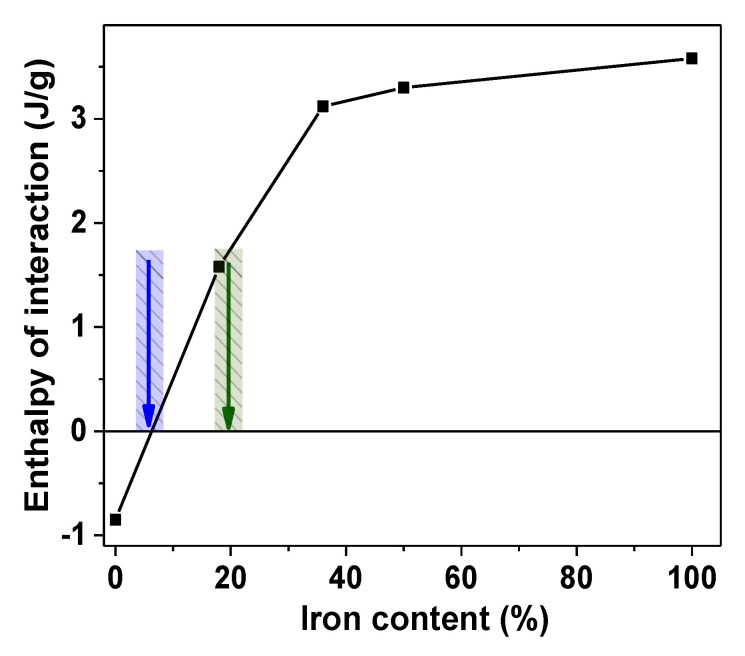
Indicative values of the enthalpy of interaction of GE polymer with zero-valent metal MNPs: Ni, Ni82Fe18, Ni64Fe36, Ni50Fe50, Fe. Blue arrow indicates zero-enthalpy of interaction composition and green arrow indicates the composition with maximum magnetic permeability (at about 79% nickel).

Figure 4 shows a plot for these values for GE/MNPs composites. They were taken from Δ*H_comp_*(ω) dependences (Figure 3b) as maximum values at the plots for GE composites with Fe, Ni50Fe50, Ni64Fe36, Ni82Fe18 MNPs and minimum value at the plot for GE/Ni composite.

The trend in Δ*H_int_* values is the same as the trend in concentration plots for Δ*H_comp_* presented in Figure 3b. Composite GE/Ni had negative value of Δ*H_int_*, while it was positive for GE/Fe composite. It is worthwhile noting that all the enthalpy changes by their definition are the difference between the enthalpy of a GE composite and the enthalpies of the components. Therefore, negative value of Δ*H_int_* means that interaction in the composite become stronger than in components. Positive value of Δ*H_int_* corresponds to the opposite. Thus, the embedding of Ni MNPs in GE composite resulted in the enhancement of interactions, which most likely occurred at the polymer/solid interface. On the contrary, positive values of Δ*H_int_* for GE/Fe composite indicated the overall weakening of interactions compared to that in the components. The opposite sign of Δ*H_int_* in GE/Ni and GE/Fe composites indicated that the mechanisms of interaction of GE polymer with the surface of Ni and Fe MNPs were different. In other words, there was a favorable adhesion of GE polymer to the surface of Ni MNPs and there was no adhesion of GE to the surface of Fe MNPs. Moreover, positive values of Δ*H_int_* in GE/Fe composites were substantial, which meant that weak interactions at GE/Fe interface likely provided the weakening of interactions in the GE polymeric matrix. All permalloy MNPs: Ni82Fe18, Ni64Fe36, Ni50Fe50 also showed positive values of Δ*H_int_*. The numerical value of Δ*H_int_* increased with the Ni/Fe ratio in permalloy. Please note that even Ni82Fe18 with the major fraction of Ni had positive enthalpy of interaction with GE. It meant that surface properties of Fe are dominant in NiFe alloys.

Magnetic nanoparticles of Ni82Fe18 composition were obtained by EEW from the wire with Ni80Fe20 composition due to the slight change of the composition in the course of fabrication. The Ni80Fe20 alloy with maximum magnetic permeability and low magnetostriction (at about 79% nickel) is the most used in sensor applications [17,18,19]. High permeability in homogeneous magnetic material appears either due to the magnetization rotation in a condition of weak crystal anisotropy or due to the displacement of the domain walls. These results can be expected for the materials with close to zero magnetostriction value [12,15,16,17]. However, the most favorable composition and particular properties of the material depend in a complex way on the preparation conditions. We, therefore, outlined the interval around Ni80Fe20 (Figure 4) emphasizing the possibility of the shift toward either higher or lower Ni content.

This experimental observation cannot for now find a reasonable explanation and further studies are needed to clarify this peculiar difference among zero-valent 3d metal nanoparticles. However, below we are making some additional comments, which could be useful for better understanding of the adhesion results.

## 4. Discussion

**Figure 5:**
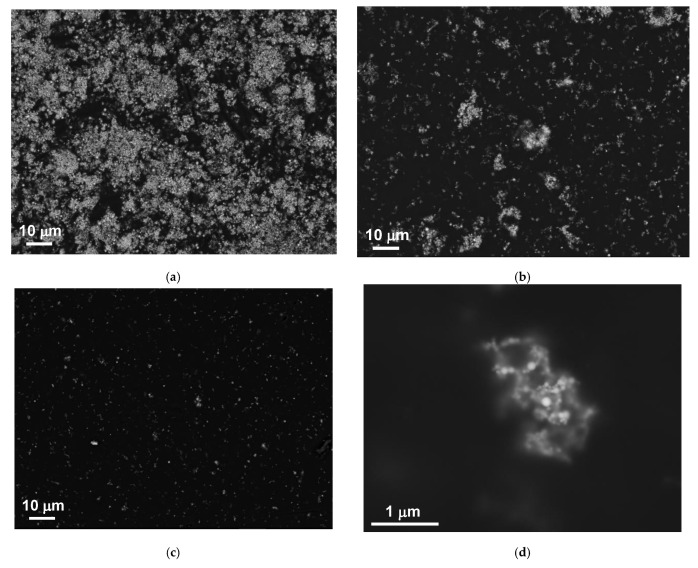
Surface properties of GE/Ni composites evaluated using scanning electron microscopy: (**a**) Ge/69 wt.% of Ni; (**b**) GE/31 wt.% of Ni; (**c**,**d**) GE/10 wt.% of Ni.

Let us now comparatively analyze some structural and magnetic properties of Ni and Ni82Fe18 GE-based composites. Backscattered electrons used for SEM evaluation [36] are high-energy electrons that are reflected or backscattered out of the volume of elastic scattering interaction with the atoms of the composite. Polymers consist of non-metallic elements with low atomic numbers and carbon is the most common element in polymer composition. Elements with the high atomic numbers scatter electrons stronger than elements with low atomic number. Therefore, the elements with the high atomic numbers appear brighter in the image offering the possibility to evaluate the contrast between areas with different chemical compositions. Figure 5 shows the surface properties of GE/Ni composites with selected concentrations of the filler. 

**Figure 6:**
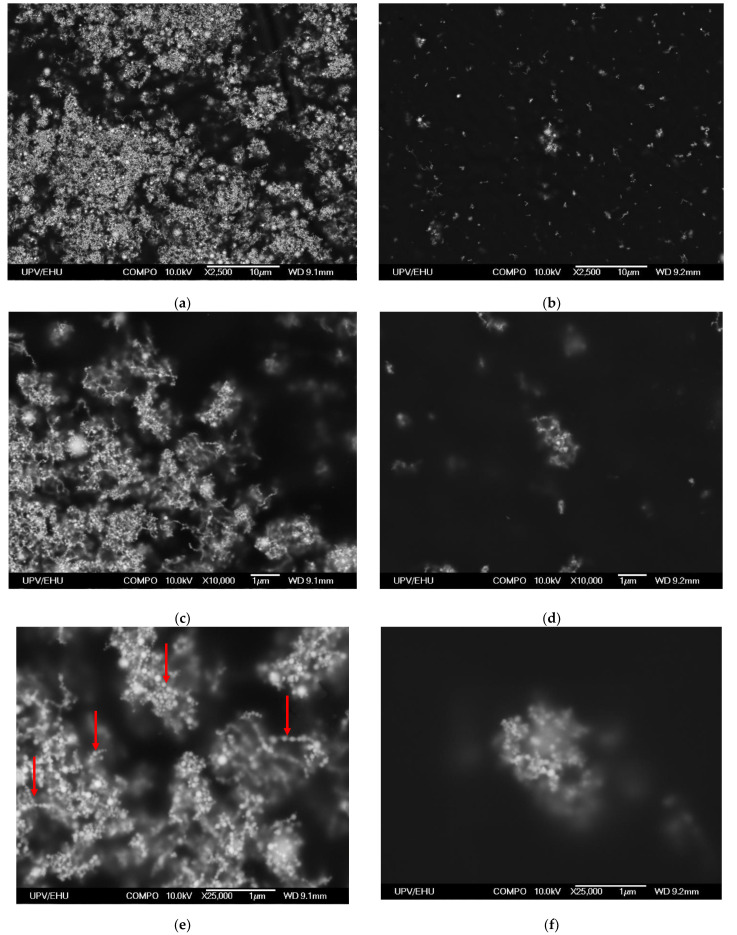
Surface properties of selected GE/Ni composites evaluated using scanning electron microscopy at different magnifications: (**a**,**c**,**e**)—GE/69 wt.% of Ni and (**b**,**d**,**f**)—GE/10 wt.% of Ni. “Chain” aggregates indicated by the red arrows.

To analyze local distribution of the aggregates and MNPs in the aggregates step-by-step increase of the magnification method was used for the analysis of the structure of the composites with high and low particles concentrations (Figure 6). One can see that at high concentrations of the MNPs the structure of the composite can be described as sufficiently uniform re-distribution of large aggregates of the order of a few microns tending to be “star”-like units with many relatively short branches. Worth mentioning the presence of the “chain”-like structures formed by at least, 7–10 particles of the medium size (Figure 6e). At low concentrations of the MNPs (even in the case of nickel MNPs with lowest saturation magnetization) the MNPs form aggregates, which are “star”-like units of the average size of the order of 1 micron or lower and turned to being spherical, i.e., the particles are not uniformly distributed over the composite even in the case when the composite preparation included all disaggregation steps. One of the reasons for such a behavior is magnetic interaction between the MNPs of this size and composition. It is also evident that “chain”-like aggregates are not a typical feature for GE composites with low concentrations of the MNPs.

Here we should come back to Table 1 and take into account available physical parameters for the comparison: *d_S_* = 53 ± 4 nm and *M_s_* = 48 ± 3 and *H_c_* = 150 ± 2 Oe values for Ni MNPs. First, the substantial coercivity and saturation magnetization value quite close to the saturation magnetization of bulk nickel confirm that the MNPs are in the multidomain state, this means they have non-zero magnetic moment in zero applied field and tend to agglomerate due to dipole-dipole interactions. As the saturation magnetization increases in the set of the materials Ni–FeNi–Fe with the increase of the iron it is to expect that the level of the dipole-dipole interactions for the particles of the same size should also increase contributing to the elevation of the level of MNPs agglomeration.

However, it is very difficult to obtain the batches of MNPs of the same size, with the same particles size distributions and similar thickness of the passivating layer. In any case, due to the difference of the composition the surface properties can vary significantly, as they depend on the type of the oxides formed onto the MNPs surfaces [37].

**Figure 7:**
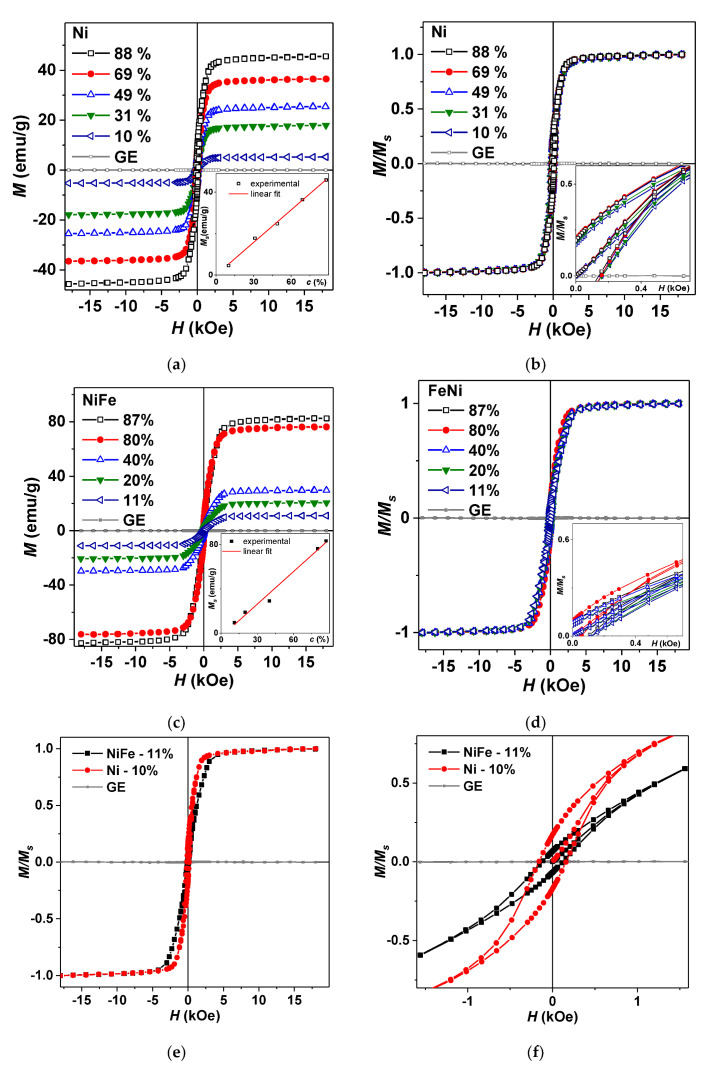
Magnetic hysteresis loops of GE/Ni (**a**,**b**) and GE/Ni82Fe18 (**c**,**d**) composites. Inset (**a**) shows the *M_s_* dependence on the concentration of Ni MNPs; inset (**b**) shows the low field part of the hysteresis loop for GE/Ni; inset (**c**) shows the *M_s_* dependence on the concentration c of NiFe MNPs; inset (**d**) shows the low field part of the *M(H)* loop for GE/Ni82Fe18 composites. Hysteresis loops of relative magnetization for GE/Ni and GE/Ni82Fe18 composites (**e**,**f**).

Let us now analyze magnetic properties of selected composites. It is logical to take a close look on GE/Ni, and GE/Ni82Fe18 composites showing (see Figure 4) opposite signs of the enthalpy of interaction between the polymer matrix and the filler—these are representative cases. Figure 7 shows magnetic hysteresis loops of GE/Ni and GE/Ni82Fe18 composites with different filler concentrations (*c*) assigned in weight %. In both cases linear dependences of *M_S_*(*c*) were observed confirming fabrication of the composites of a good quality. Although the magnetic signal of GE is diamagnetic, in all fabricated composites, it is very small in comparison with ferromagnetic responses of the nanoparticles and therefore GE magnetic contribution should not be taken into account.

To understand to what extent the concentration of nanoparticles with different compositions and their interactions affect the magnetic properties of composites the magnetic hysteresis *M*(*H*) loops were represented in *M*/*M_s_* form (Figure 7e,f). One can see that in most general sense all *M*/*M_s_* hysteresis loops of each type of the composites have very similar shape. Direct comparison of the *M*/*M_s_* values for GE/Ni and GE/Fe18Ni82 composites with low concentration of the MNPs showed that they are quite similar. The same is true for the values of the remnant magnetizations, *H_c_* values and even the field dependences of primary magnetization curves. Comparative analysis of M/M_s_ magnetic hysteresis loops of GE/Ni (*c* = 10 wt.%) and GE/Ni82Fe18 (*c* = 11 wt.%) composites shows their similarity. Even so, in the small magnetic fields *M*/*M_s_* parameter increases much faster and coercivity is higher in the case of nickel composite. This can be a consequence in the average size of the particular batch (Table 1): magnetization of the larger nanoparticles includes processes that are more complex and for the whole ensemble requires an application of the higher magnetic field to start.

As mentioned before the Ni80Fe20 alloy in the shape of thin films, had found many technological applications in the area of inductors and magnetic field sensors [18,19]. In recent years, the FeNi films deposited onto flexible substrates attracted additional interest [37,38,39,40,41]. However, in most of the cases the functional properties of FeNi thin films or multilayered structures based on FeNi components are lower in comparison with the same structures deposited onto rigid substrates. One of the reasons for observed behavior is poor adhesion of the metallic film onto the surface of flexible substrate (such as Kapton, polyester, cyclo olefin copolymer, and others). One of the strategies to improve adhesion was the deposition of the appropriate buffer layers (Al, Cu, Ti) [36,38] in combination with usage the multilayered structures favoring the stress relaxation [39,42,43].

In this study, in the case of GE/Ni composites with Ni MNPs as a filler, we had observed a favorable adhesion of GE polymer to the surface of Ni MNPs. This means that deposition of nickel layer onto polymer substrate might be the useful technological step and probably the solution of the well-known problem, at least this research direction seems to be interesting to follow. In addition, the microwave properties of Fe, FeNi, Ni, and NiCo MNPs of polymer-based composites can be mentioned. GE varnish was previously used for many nanostructured materials high frequency characterization due to very low contribution of the matrix itself and simplicity of the composite preparation [44,45]. Apart from usual applications as microwave absorbers [46,47] these composites were tested as model materials for the development of magnetic biosensors [48].

It is also important to attract attention to the strategy of the analysis of the surface properties of nanoparticles during their interactions with polymers for the prediction of the functional properties of thin films and multilayered structures deposited onto flexible substrates. As the effective surface available for the interaction of metallic film and polymer substrate is rather small, the only way to find appropriate combinations of thin film and polymer is multiple searches for the best deposition conditions. However, usage of the MNPs of requested composition and selected polymer for evaluation of their adhesive properties by existing methods of physical chemistry might be very useful complementary way to solve the problem.

## 5. Conclusions

Magnetic nanoparticles of various compositions were fabricated by EEW. The results of their structural and magnetic characterization were comparatively analyzed. The average size of all kinds of MNPs was in the interval of about 50 to 100 nm. All MNPs were in the multidomain state with the saturation magnetization slightly reduced in comparison with *M_s_* value for corresponding composition. However, the observed reduction was explained in the framework of scaling laws for MNPs and based on the surface oxidation. Zero-valent nickel, iron, and permalloy MNPs were embedded into polymeric matrix based on GE varnish. Chemical nature of the GE varnish had been analyzed using IR spectroscopy. The main component of the GE polymer is self-associated poly(vinyl butyral). The interaction of GE polymer with zero-valent metal magnetic nanoparticles was studied by microcalorimetry. The positive values of the enthalpy of interaction with GE increase in the series Ni82Fe18, Ni64Fe36, Ni50Fe50, and Fe. Meanwhile, interaction of Ni MNPs with GE polymer was characterized by the negative change in the enthalpy. It meant that there is no interfacial adhesion of GE polymer to the surface of Fe and permalloy MNPs in composites.

Structural evaluation of the GE/Ni composites with the MNPs having the lowest saturation magnetization confirmed that despite the special efforts to separate MNPs in the course of fabrication of the composite they tended to be aggregated even for the materials with lowest MNPs concentrations. “Chain”-like aggregates were not typical for GE composites with low concentrations of the MNPs. At high concentrations of the Ni MNPs aggregates of the order of a few microns tending to be “star”-like units with the presence of the “chain”-like structures were observed. One of the reasons for such a behavior is a magnetic interaction between the MNPs.

To understand to what extent the concentration of MNPs and their interactions affect the magnetic properties M/Ms hysteresis loops were analyzed for GE/Ni and GE/NiFe composites with different concentration of the filler. Direct comparison of the M/Ms values for GE/Ni and GE/Fe18Ni82 composites with low concentration of the MNPs confirmed their similarity. It was shown that Ni95Fe5 composition might be interesting for future investigations, as the enthalpy of interaction with GE polymer was close to zero for it. In the case of GE/Ni composites with Ni MNPs as a filler, a favorable adhesion of GE polymer to the surface of Ni MNPs was observed. The deposition of nickel layer onto polymer substrate might be useful technological step for fabrication of FeNi films with enhanced functional properties when deposited onto flexible substrates.

## Figures and Tables

**Table 1:** Selected characteristics of EEW metallic nanoparticles: Specific surface area—*S_sp_*, density—ρ, apparent average diameter—*d_S_*, phase composition (all phases are cubic); saturation magnetization *M_s_* and coercivity *H_c_* measured for nanoparticles at 20 °C.

Mark	Description	*S_sp_* (m^2^/g)	*ρ* (g/cm^3^)	*d_S_* (nm)	Phase Composition	*M_s_* (emu/g)	*H_c_* (Oe)
Fe	Iron	7.5	7.8	102 ± 8	98% (S.G: Fm-3m) fcc, 0.35927(2) 2% (S.G.: I m-3m) bcc a = 0.2862(3)	200 ± 5	300 ± 5
Ni50Fe50	Permalloy: 50% Ni, 50% Fe	12.5	8.4	62 ± 5	100% (S.G.: F m-3m) bcc, a = 0.3569(1)	140 ± 5	180 ± 2
Ni64Fe36	Permalloy: 64% Ni, 36% Fe	12.6	7.8	61 ± 5	90% (S.G: Fm-3m) fcc, a = 0.3592(2) 10% (S.G.: I m-3m) bcc, a = 0.2862(3)	110 ± 5	160 ± 2
Ni82Fe18	Permalloy: 82% Ni, 18% Fe	8.0	8.4	86 ± 7	100% (S.G: Fm-3m) bcc, a = 0.3548(1) nm	82 ± 5	100 ± 1
Ni	Nickel	12.6	8.9	53 ± 4	100% (S.G: Fm-3m) bcc, a = 0.3524(2) nm	48 ± 3	150 ± 2

## Data Availability

Data available from the corresponding author on reasonable request.
